# Biofortification of common bean (*Phaseolus vulgaris* L.) with iron and zinc: Achievements and challenges

**DOI:** 10.1002/fes3.406

**Published:** 2022-06-30

**Authors:** Raul Huertas, Barbara Karpinska, Sophia Ngala, Bertha Mkandawire, Joyce Maling'a, Elizabeth Wajenkeche, Paul M. Kimani, Christine Boesch, Derek Stewart, Robert D. Hancock, Christine H. Foyer

**Affiliations:** ^1^ Environmental and Biochemical Sciences The James Hutton Institute Dundee UK; ^2^ School of Biosciences, College of Life and Environmental Sciences University of Birmingham Edgbaston UK; ^3^ Department of Plant Science and Crop Protection, College of Agriculture and Veterinary Sciences University of Nairobi Nairobi Kenya; ^4^ The Food, Agriculture and Natural Resources Policy Analysis Network (FANRPAN) Pretoria South Africa; ^5^ Kenya Agriculture and Livestock Research Organization (KALRO) Food Crops Research Institute Kitale Kenya; ^6^ School of Food Science and Nutrition University of Leeds Leeds UK; ^7^ School of Engineering and Physical Sciences Heriot‐Watt University Edinburgh UK; ^8^ Cell and Molecular Sciences The James Hutton Institute Dundee UK

**Keywords:** bioavailability, biofortification, breeding, common bean, *Phaseolus vulgaris*

## Abstract

Micronutrient deficiencies (hidden hunger), particularly in iron (Fe) and zinc (Zn), remain one of the most serious public health challenges, affecting more than three billion people globally. A number of strategies are used to ameliorate the problem of micronutrient deficiencies and to improve the nutritional profile of food products. These include (i) dietary diversification, (ii) industrial food fortification and supplements, (iii) agronomic approaches including soil mineral fertilisation, bioinoculants and crop rotations, and (iv) biofortification through the implementation of biotechnology including gene editing and plant breeding. These efforts must consider the dietary patterns and culinary preferences of the consumer and stakeholder acceptance of new biofortified varieties. Deficiencies in Zn and Fe are often linked to the poor nutritional status of agricultural soils, resulting in low amounts and/or poor availability of these nutrients in staple food crops such as common bean. This review describes the genes and processes associated with Fe and Zn accumulation in common bean, a significant food source in Africa that plays an important role in nutritional security. We discuss the conventional plant breeding, transgenic and gene editing approaches that are being deployed to improve Fe and Zn accumulation in beans. We also consider the requirements of successful bean biofortification programmes, highlighting gaps in current knowledge, possible solutions and future perspectives.

## Graphical abstract

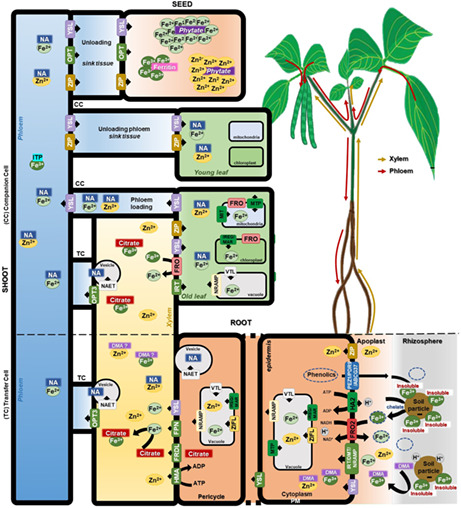

## 1 INTRODUCTION

Future projections for population and climate change give cause for concern in terms of increased food insecurity. The United Nations (2019) predicts that although the increase in population growth is slower than at any time since the 1950s, the 2019 projection continues to suggest that the global population will be about 8.5 Bn, 9.7Bn and 10.9Bn in 2030, 2050 and 2100, respectively. Despite the beneficial carbon fertilisation effect of rising global CO_2_ concentrations, associated changes in weather patterns will have variable impacts on crop productivity and nutritional quality highlighting the need to limit climatic changes induced by human activity.

The Intergovernmental Panel on Climate Change (2018) and others (Fawzy et al., 2020) have recommended that significant behavioural changes are needed in all sectors of life if global emissions are to be reduced to limit global temperature increases to <2°C by 2100. The adverse consequences of climate change such as storms, floods, wildfires and drought are likely to affect ~68 million people and create economic losses of about $131 billion (Fawzy et al., 2020). These changes will severely impact on the global food system, particularly agriculture, which is itself a major emitter (21%–37%) of greenhouse gases (GHSs; Mbow et al., 2019). Climatic instability will inevitably lead to decreased crop productivity. Irregular and/or extreme rain patterns will be particularly problematic for crop production in developing nation (Malhi et al., 2021). Developing countries are most affected by global temperature rise. As an example, an increase of 1°C results in serious changes in yield losses were consistently across 5 global sites and ranged between 3.1% and 7.1% for soybean across countries (Zhao et al., 2017).

Elevated atmospheric CO_2_ (eCO_2_) levels tend to have a negative impact on the protein content of cereals and vegetable crops and increase secondary metabolites such as flavonoids and ascorbic acid (Dong et al., 2018; Halford et al., 2015). Crucially, growth under eCO_2_ results in reduced grain legume Fe and Zn contents (Köhler et al., 2019; Myers et al., 2014) although elevated temperatures decreased the negative impact of eCO_2_ on grain legume Fe and Zn levels (Köhler et al., 2019). While drought decreased the Fe levels of common beans, the Zn content was increased together with phytic acid, a key antinutrient that adversely affects Zn bioavailability (Hummel et al., 2018; Losa et al., 2022).

At a global level the interplay between agriculture, climate change, GHG emissions, food security and nutrition has sparked many debates such as conventional versus regenerative agriculture, circular versus linear production chains and livestock versus plant‐based foods. None of these are clear cut but the potential of plant‐based foods, both in minimising the environmental impact of agriculture as well as in providing an inexpensive source of appropriate nutrition, especially in developing countries, cannot be understated.

## 2 IRON AND ZINC DEFICIENCY

Access to dietary Fe and Zn is strongly influenced by economic circumstances and dietary patterns. For example, haem Fe, available from animal‐based foods, is better absorbed (15%–40%) than non‐haem Fe from plant‐based foods (1%–15%; Shubham et al., 2020). Vegan diets, consumer preferences and limited meat availability (e.g. developing countries), dictate that an adequate intake of Fe and Zn must come from plant‐based foods. Significant deficiencies in these minerals are common in developing countries (Joy et al., 2014; Ohanenye et al., 2021; Wessells & Brown, 2012) where they not only have impacts on human health and well‐being but also negatively impact developing economies. For example, undernutrition defined as both insufficient intake of protein and calories as well as deficiencies in micronutrients represent economic losses averaging 11% of GDP across Africa (International Food Policy Research Institute, 2016). Hence, strategies to enhance Fe and Zn accumulation in plants as well as their bioavailability to enhance effective absorption by the human body, are key targets for crop improvement worldwide. To address these issues, international consortia such as *HarvestPlus*, *HarvestPlus Latin American and Caribbean (LAC)* and the *Pan Africa Bean Research Alliance (PABRA)* seek to increase the Fe and Zn levels of beans growing in East Africa, South Asia and Latin America (Blair et al., 2010, 2013, 2021; Herrington et al., 2019; Kimani & Warsame, 2019; PABRA, 2017).

**FIGURE 1:**
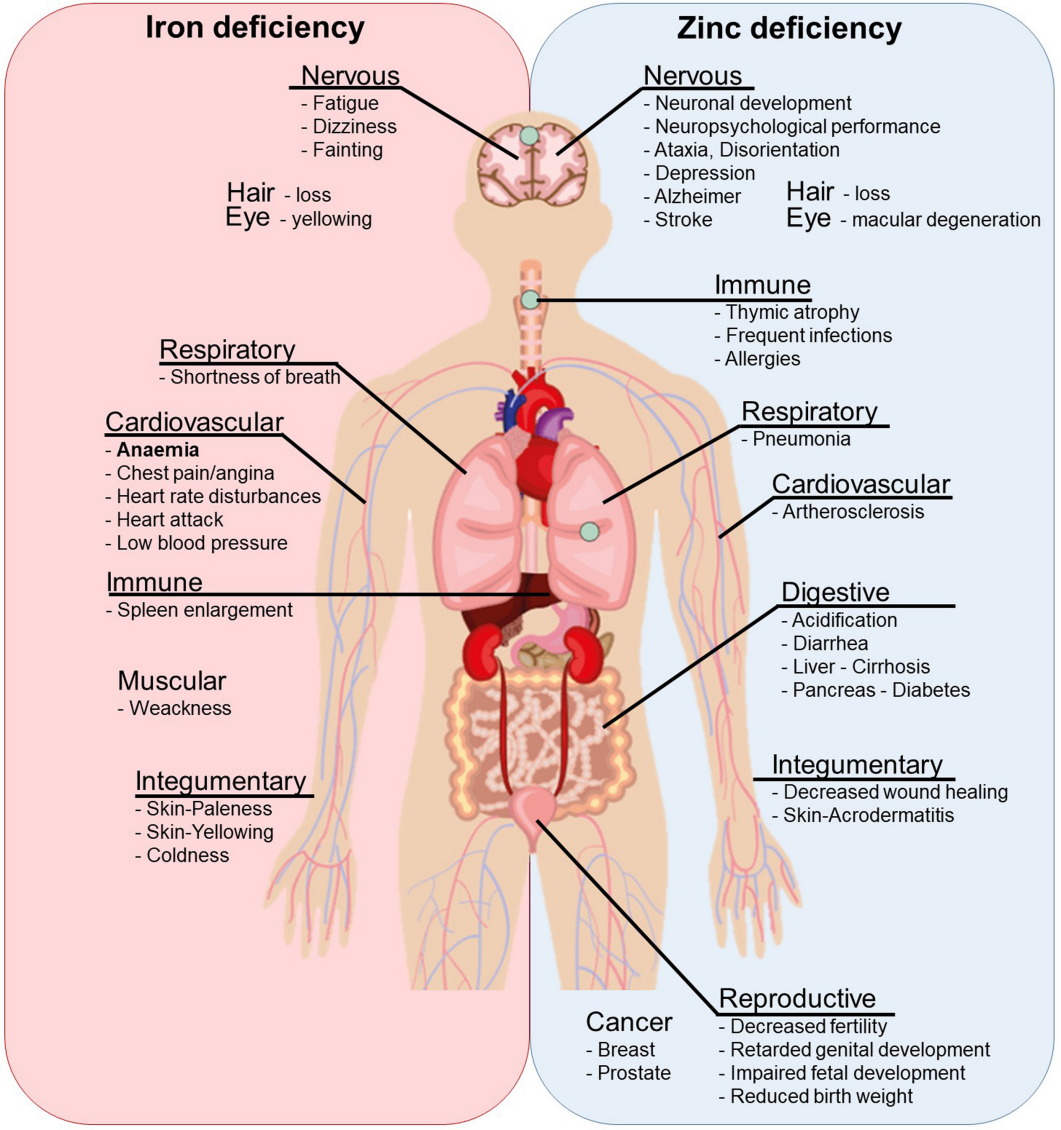
Overview of symptoms and diseases associated with imbalanced iron (left) and zinc (right) homeostasis in the human body

**FIGURE 2:**
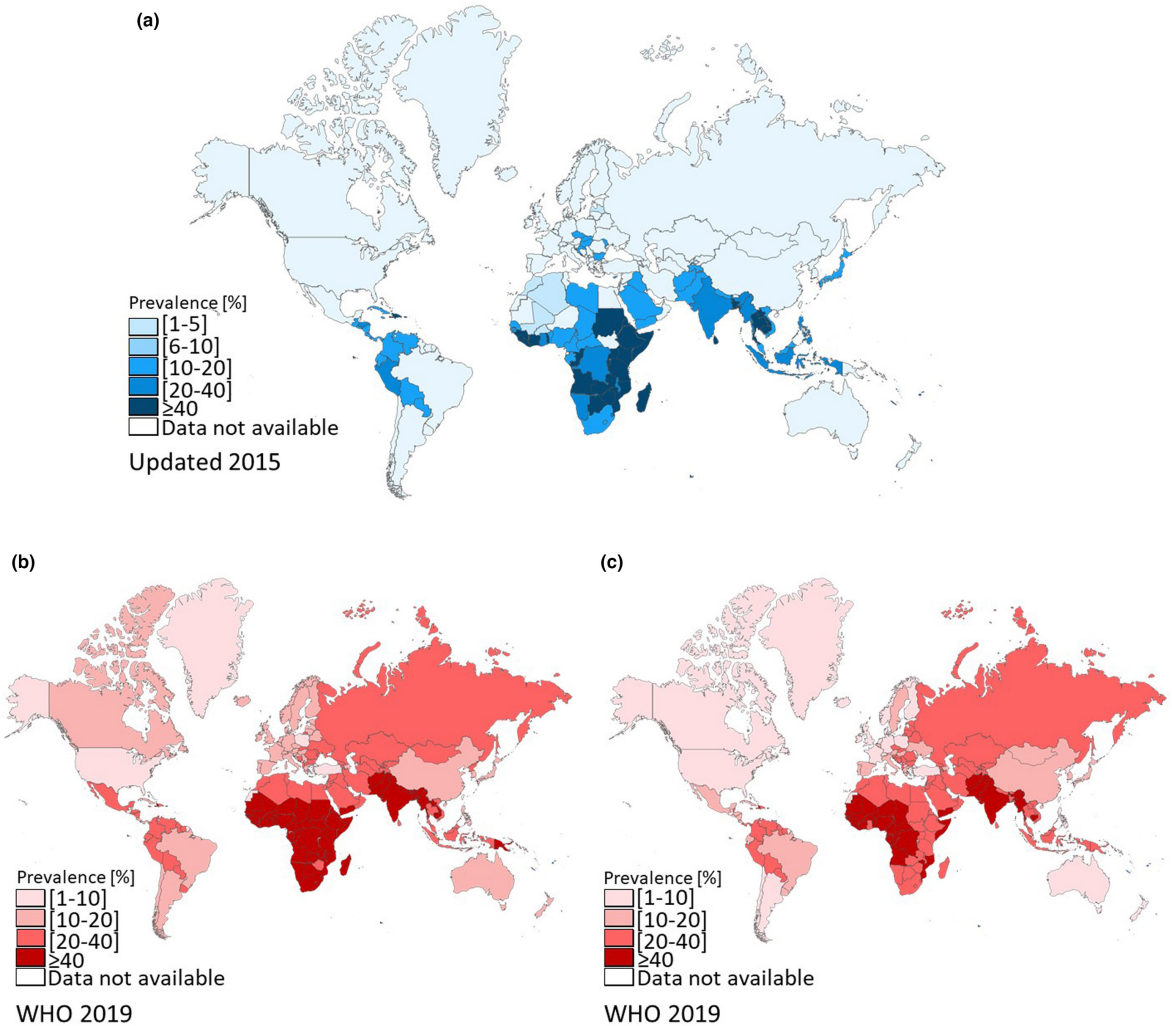
World map showing the prevalence of iron and zinc deficiencies as the percentage of population with intakes below physiological requirements for each country. (a) Prevalence of zinc deficiency. Map generated from Wessells and Brown (2012) and updated according to Joy et al. (2013) and Kumssa et al. (2015). (b) Prevalence of anaemia associated mainly to iron deficiency among preschool‐age children (6–59 months). (c) Prevalence of anaemia among women of reproductive age (15–49 years). Map generated from WHO data (2019); accessed September 2021

Deficiencies in Fe and Zn affect people of all ages. However, their effects are greatest in pregnant women and children, especially young infants. The Zn requirement for adults ranges from 8 to 11 mg/day but pregnant and lactating women require 11 to 13 mg/day. The requirement for Fe is greater, ranging from 12 to 28 mg/day for most adults, increasing from 30 to 38 mg/day for pregnant and lactating women (Dietary Reference Intakes, 2019). Micronutrient deficiencies (Hidden Hunger) caused by inadequate dietary intake, excessive losses or malabsorption led to a range of pathologies including anaemia, several chronic diseases, weakened immunity and delayed development (Lopez et al., 2016; Maggini et al., 2018; Philipo et al., 2021; Stammers et al., 2015; Figure 1). Although often less apparent than starvation or protein deficiency, Fe and Zn deficiency is common in less developed countries, including in sub‐Saharan Africa and Central and South America (Gupta et al., 2020; Muthayya et al., 2013; Rehman et al., 2020; Figure 2).

Zn deficiency plays a part in 18%–22% of lower respiratory tract infections, 11%–13% diarrheal diseases and 10%–22% malaria (Philipo et al., 2021; WHO, 2013). Fe deficiency (including anaemia) is similarly debilitating where it accounts for a total of 58.6 (40.14–81.1) million years lived with disability in 2019 (Gardner & Kassebaum, 2020). Moreover, the highest burden was experienced in Western Sub‐Saharan Africa, South Asia and Central Sub‐Saharan Africa. Fe deficiency and anaemia are especially prevalent in children and pregnant women in Africa, leading to impaired cognitive and physical development and stunting in children, as well as a reduction in the reproductive capacity of adults (Gupta et al., 2020; Mwangi et al., 2017; Mwangi et al., 2021). The 2011 Kenya National Micronutrient Survey showed that Fe deficiency and Fe deficiency anaemia in pregnant women were present at 36.1% and 26%, respectively, and in pre‐school children at 21.8% and 13.3%, respectively. Even non‐pregnant women had a prevalence of 21.3% for Fe deficiency and 14% for anaemia (Kenya National Micronutrient Survey, 2011; Young, 2018). Fe deficiency in the pre‐school population living in 60 villages in Western Kenya and North Rift Valley was reported to be 46% (in the year 2012) and 67% (in the year 2021), respectively (Grant et al., 2012; Oyungu et al., 2021). The 2011 National Micronutrient Survey also found that 83.3% of pre‐school children, 82.3% of non‐pregnant women, 80.2% of school going children, 74.8% of men and 68.3% of pregnant women suffered from Zn deficiency.

## 3 COMMON BEANS

Common bean (*Phaseolus vulgaris* L.) is the most consumed food grain legume worldwide, and dried beans are the most produced in developing countries (Gregory et al., 2017; Nassary et al., 2020; Rawal & Navarro, 2019). Land dedicated to bean production is ~33 M hectares globally, with 7.9 M hectares in Africa alone (FAOSTAT, 2019; Table 1). An estimated 5 million hectares are grown by smallholder farmers in Africa (mainly women) in sole stand and in association with other legumes, cereals, root crops and tree crops. Beans deliver dietary protein for >300 million people in rural and urban Eastern African and Latin American communities (Buruchara et al., 2011; Petry et al., 2015). Indeed, bean is a primary source of protein and micronutrients especially Fe and Zn for over 200 million people in rural and poor urban communities in Africa, who can hardly afford alternative sources of these nutrients on a regular basis. The average consumption of common beans in Latin America ranges 10–18 kg per person per year, whereas in East Africa the consumption can be as high as 50 kg per person annually (Celmeli et al., 2018; Katungi et al., 2009). The highest yields (averaging 2000 kg/ha) have been achieved in Europe, which has only a small proportion of global production by area (Table 1). Yields in Africa are only half those obtained in Europe, largely because of poor soils, in which key microelements are in short supply. Hence, there are variations in productivity across Africa. Northern Africa achieves yields of 4 tonnes per hectare while the yield in Middle Africa are only 0.68 tonnes per hectare (FAOSTAT, 2019). The effects of drought and heat associated with the global climate change on common bean production and mineral content have recently reviewed by Losa et al. (2022).

**TABLE 1:** Harvested area, yield and total production of dry beans by continent and worldwide

	Area harvested		Yield		Total production	
	Mha	%	kg/ha	%	Mt	%
Europe	0.2	0.6	1806.1	33.3	0.4	1.3
Asia	18.3	55.5	783.6	14.5	14.4	49.7
Americas	6.5	19.8	1075.7	19.9	7.0	24.4
Africa	7.9	23.9	893.4	16.5	7.1	24.4
Oceania	0.1	0.3	857.7	15.8	0.1	0.3
Worldwide	33.1		5416.5		28.9	

*Source*: Food and Agriculture Organization Statistical Databases (FAOSTAT) was used to develop this table (Data: 2019; Accessed: September 2021).

Mha = a million of hectares (ha); Mt = megatonne, a million of metric tonnes (tonnes).

Beans have a high nutritive value due to the balance of carbohydrates to proteins, and a high amino acid diversity compared to cereals (Sa et al., 2020). At 340 calories per 100 g, beans not only provide energy, but they can also contribute up to 35% of daily protein requirements. Beans also contain vitamins, dietary fibre and high concentrations of micronutrients. For example, common bean seeds have 4–10 times more Fe and 2–3 times more Zn than cereals such as maize, wheat and rice (Blair, 2013; Welch & Graham, 2004). Bean Fe levels are generally in the range of 61–71 mg/kg. However, values as low as 34 mg/kg or as high as 152 mg/kg have been reported in some genotypes. Similarly, average Zn content ranges from 28 or 31 mg/kg, with extremes of 18 and 77 mg/kg (Beebe, 2020; Caproni et al., 2020; Glahn & Noh, 2021; Gunjaca et al., 2021; Katuuramu et al., 2021; Murube et al., 2021).

Countries of sub‐Saharan Africa will account for more than half of the growth of the world's population between 2019 and 2050, increasing the necessity for a sustainable provision of nutritious food. Common bean has an important role to play in this regard and it is thus likely to become an increasingly significant food source in Africa where they are predicted to play an essential role in nutritional security (de Valença et al., 2017; Moloto et al., 2018; Philipo et al., 2021). To meet increases in demand, annual yields have been increasing by over 2% per year between 2006 and 2018 in primary African production centres in Eastern, Southern and Western Africa. This translates to annual production increases as high as almost 8% in Western Africa, where yield increases have been matched by significant expansion (>5% per annum) of the production area (Farrow & Muthoni‐Andriatsitohaina, 2020).

African soils are often low in essential nutrients, particularly Zn (Hengl et al., 2017). Correlations between soil and leaf Zn levels have been reported for a range of staple crops (Kihara et al., 2020). Although Fe is not a major micronutrient that limits crop yields, significant variations in the Fe contents of staple crops have been reported and these have been linked to soil Fe availability (Gashu et al., 2021). Soil Fe status can therefore be an important factor in dietary availability. Fertilisation with Zn‐based fertilisers at a time that ensures grain Zn enrichment can be cost‐effective and efficient (De Groote et al., 2021; Praharaj et al., 2021). A number of assessments and programmes for bean improvement seeking to enhance the levels of these micronutrients (Beebe et al., 2000) have been undertaken in recent years (Assefa et al., 2019; Mukankusi et al., 2019). Indeed, an extensive international programme for the development and dissemination of micronutrient dense bean varieties in Africa started in 2004. The programme was supported by the *Association of Strengthening Agricultural Research in East and Central Africa (ASARECA)* through the East and Central Africa Bean Research Network (ECABREN). ASARECA is a sub‐regional organisation comprising of 11 member countries: Burundi, Democratic Republic of Congo, Ethiopia, Eritrea, Kenya, Madagascar, Tanzania, Rwanda, Sudan, South Sudan and Uganda. The programme was based at the College of Agriculture and Veterinary Sciences, University of Nairobi in Kenya.

Moreover, there has been an increased focus on the bioavailability of Fe and Zn, and how it is affected by food preparation approaches such as soaking, boiling, roasting, dehulling, germination, fermentation, supplementation with various chemicals and enzymes and, more recently, extrusion cooking (Kinyanjui et al., 2015). Indeed, the *International Center for Tropical Agriculture (CIAT)* in consultations with nutritionists established a breeding goal level of 94 mg/kg Fe above the value of a standard local variety to achieve 30% of average daily Fe requirement, assuming 7% bioavailability, 90% retention after cooking, and a high level of consumption of 200 g/day for adults and 100 g/day for children (Beebe, 2020).

## 4 PLANT IRON AND ZINC REQUIREMENTS

Fe and Zn are essential plant cofactors with key roles in important plant processes such as photosynthesis, respiration and stress tolerance (Rout & Sahoo, 2015; Sharma et al., 2013; Tripathi et al., 2018). In addition, Fe‐ and Zn‐dependent processes are crucial for the establishment of endosymbiotic associations with arbuscular mycorrhiza and with soil rhizobia in legume nodules (Day & Smith, 2021; Gonzalez‐Guerrero et al., 2016). Fe deficiency leads to chlorosis and decreased vegetative growth, resulting in poor crop yield and quality. In comparison, plants have a relatively low requirement for Zn, even though it is an important micronutrient being an essential component of enzymes such as Cu, Zn superoxide dismutase and required for processes such as auxin metabolism and chlorophyll synthesis (Sharma et al., 2013).

While Fe‐deficiency is crucial to plant productivity, excess Fe is equally problematic, particularly in flooded soils, in which Fe is present mainly as soluble Fe^2+^ due to the low redox potential that prevails under anaerobic conditions and low pH. Fe toxicity is a serious stress that adversely affects the growth of crops such as wetland rice in Asia and West Africa (Sahrawat, 2005). Excess soil Fe availability can impair the acquisition of other nutrients (Leskova et al., 2021; Xue et al., 2016). Hence, cellular Fe homeostasis is tightly controlled. Fe deposits at the root surface can form a physical barrier to prevent unnecessary Fe uptake, while reduced Fe translocation from roots to shoots and storage of Fe in different sub‐cellular compartments, particularly in the apoplast and vacuoles, are thought to alleviate Fe toxicity. The main function of the ferritin proteins, which can store up to 4000 Fe atoms, is to limit cellular Fe concentrations to levels (10^−3^–10^−5^ M) that are commensurate with metabolic functions (Briat et al., 2010). In addition, ferritin also plays an important role in the defence against pathogens (Aznar et al., 2015).

**FIGURE 3:**
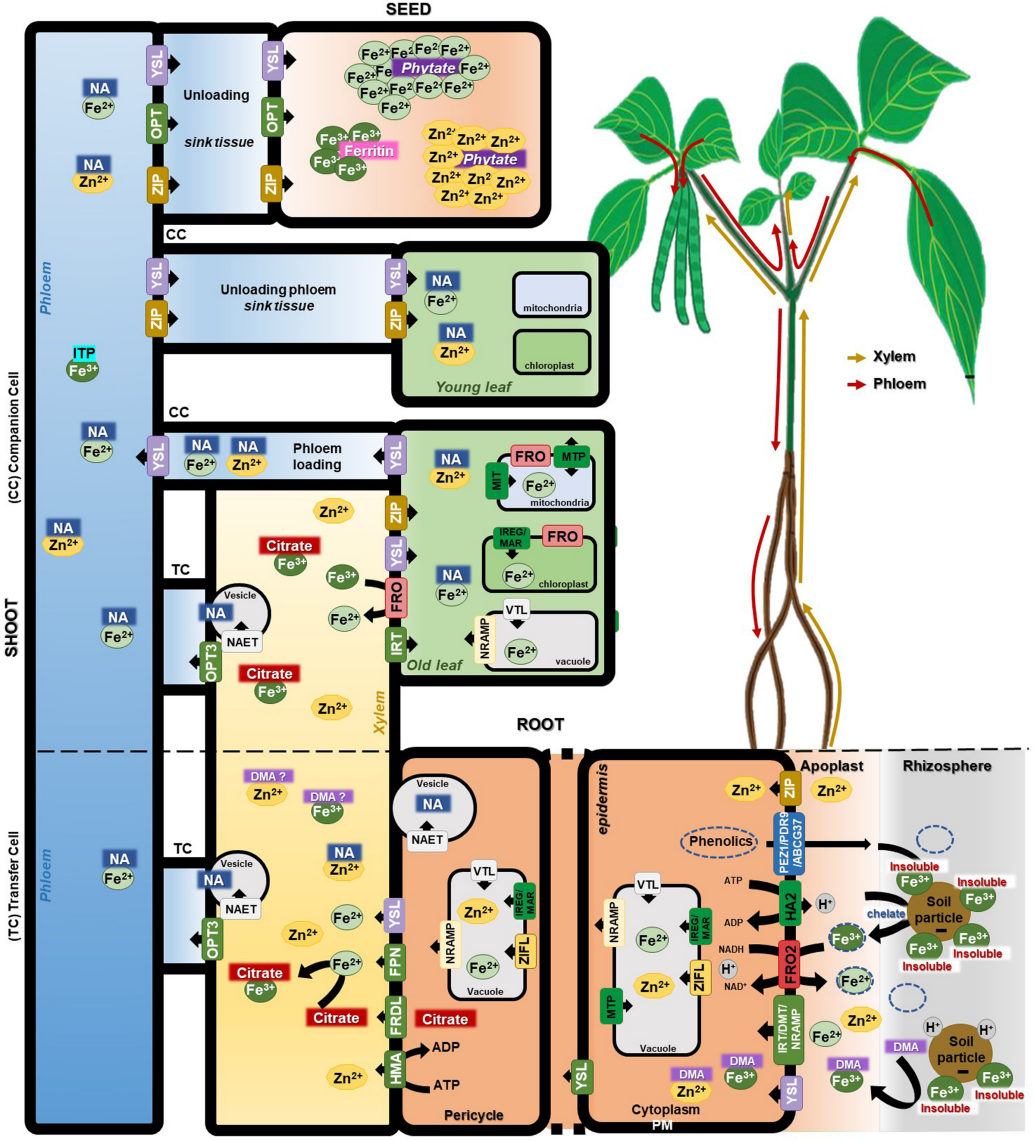
Schematic diagram of a mechanistic model representing current knowledge about the physiological processes, genetic elements and minerals movement involved in Fe and Zn homeostasis in common bean. Source–sink relationships are represented according to the detailed information provided in the main text: Uptake strategies from rhizosphere and mobilisation into the root cells, root‐to‐shoot translocation via xylem, mineral partitioning to the phloem and seed mineral loading, including intracellular compartmentalisation

There have been a number of excellent recent reviews describing Fe and Zn metabolism and translocation in plants, together with the associated regulatory mechanisms (Balafrej et al., 2020; Curie & Mari, 2017; Dey et al., 2020; Gao & Dubos, 2021; Rai et al., 2021; Ram et al., 2021; Rehman et al., 2021; Sperotto et al., 2018; Whitt et al., 2020; Yadav et al., 2021). Such reviews often focus on cereal crops such as wheat, rice and barley (Detterbeck et al., 2020; Huang et al., 2020; Pandit et al., 2021; Pradhan et al., 2020; Xia et al., 2020). Despite this extensive knowledge, relatively little information is available on these processes in legumes, particularly common bean. We have summarised current knowledge in Figure 3, which provides a schematic model of the regulation of Fe and Zn homeostasis in plants, highlighting findings in legumes and beans. There are a number of possible rate‐limiting steps for Fe and Zn accumulation in seeds, which are potential targets for biofortification. These are Fe and Zn uptake from soil and transport from the root, the storage of Fe and Zn in photosynthetic and metabolic proteins, and their eventual remobilisation at grain filling. These processes also require a knowledge of the loading and transport processes in the phloem and unloading processes in the seed. All of these processes are likely to require the input and regulation of multiple genes, many of which are yet to be identified, particularly in legumes (Roorkiwal et al., 2021; Sperotto & Ricachenevsky, 2017).

## 5 UPTAKE FROM RHIZOSPHERE AND MOBILISATION INTO THE ROOT CELLS

Plants absorb mineral nutrients through root cells and root system architecture (RSA), the spatial three‐dimensional roots arrangement, determines the active root surface area and allows the exploration and uptake of mineral nutrients from the soil (Li et al., 2016). Reduced soil nutrient availability leads to specific RSA phenotypes. In general, limited Fe availability inhibits lateral root length and slightly reduces branching, while Zn limitation also inhibits lateral root growth but stimulates branching (Shahzad & Amtmann, 2017).

Plants have evolved two main strategies for uptake of Fe using a combination of reduction and chelation mechanisms (Connorton et al., 2017). Dicotyledonous and non‐graminaceous plants utilise strategy I, which requires the combined action of specific enzymes and transporters to mobilise soil nutrients making them available for uptake by roots. Root plasma membrane H^+^‐ATPase (HA2‐like) family members increase the solubility of insoluble ferric ions (Fe^3+^) by acidification of the rhizosphere in the immediate vicinity of the roots. Fe^3+^ is then reduced by the activity of apoplastic ferric reduction oxidase (FRO2‐like) prior to transport across the epidermal cell membranes. Fe^2+^ is translocated by two classes of Fe^2+^ transport protein families: ZIP [Zinc resistance transporter (ZRT)/Iron‐Regulated Transporter (IRT) – Related Protein] and DMT/NRAMP [Divalent Metal ion Transporter 1 (DMT)/Natural Resistance Associated Macrophage Protein 1 (NRAMP)] (Roorkiwal et al., 2021). Induction of these molecular components is accompanied by changes in root morphology and architecture, particularly increases in secondary and lateral roots, absorbent hairs and transfer cells (Fukao et al., 2011; Reyt et al., 2015). The intracellular Fe and Zn trafficking and subcellular homeostasis in organelles have been recently reviewed by Vigani et al. (2019) and Przybyla‐Toscano et al. (2021) showing that different members of the same family (e.g. FRO) have specific roles in subcellular compartments in addition to an organ‐specific role. The expression of the FRO, ZIP and NRAMP genes is controlled by the Fe content of the soil and is also regulated by different transcription factors, as discussed later in this review.

Fe and Zn move through the root via symplastic and/or apoplastic pathways. Metabolites such as citrate and nicotianamine (NA), and also histidine and glutathione have been implicated in this transport. Such metabolites act as chaperones limiting precipitation and potential damage (Clemens, 2019; Curie et al., 2009). Free Fe is thought to form Fe^3+^‐citrate, Fe^3+^‐NA and Fe^2+^‐NA complexes in the cytoplasm of dicotyledonous plants. In contrast, monocotyledonous species utilise strategy II (chelating strategy) in which phytosiderophore (PS) nicotianamine (NA), which bind Fe^3+^, are released into the rhizosphere (Kobayashi et al., 2010). The Fe^3+^‐PS complexes are taken up by specific transporters belonging to the YS1/YSL [YELLOW‐STRIPE 1 (YS1)/YS1‐LIKE (YSL)] family (Schaaf et al., 2004; Xiong et al., 2013). The YSL3 and YSL1 proteins are strongly expressed in the leaf vasculature where they function to take up Fe that arrives in leaves via the xylem (Waters et al., 2006). In the following discussion, we will focus on the genes and processes that have been identified and characterised in common bean.

The common bean genome contains putative homologues for all the components of uptake and chelation. This includes genes encoding several FRO proteins and up to 13 HA2‐like proteins (Table S1). Three putative FRO‐like genes are associated with Fe and Zn content in beans (Izquierdo et al., 2018). However, they have little homology to the Arabidopsis FRO2 genes and are currently annotated as Cytochrome b561/ferric reductase transmembrane proteins (Table S1). Attempts have been made to identify IRT1 homolog genes in common bean. The H^+^‐ATPase and IRT1 proteins accumulate in root nodules under Fe deficiency (Slatni et al., 2012), furthermore Castro‐Guerrero et al. (2016) described an IRT1‐like gene that acts as a ZIP2 Zn transporter (Astudillo‐Reyes et al., 2015). Several NRAMP genes have been identified in legumes, with 13 members in soybean (Qin et al., 2017) and seven in *Medicago truncatula* (Tejada‐Jimenez et al., 2015). While only one NRAMP gene has been reported in peanut (Xiong et al., 2012), seven NRAMP genes have been identified in common bean. Analysis of their tissue‐specific expression patterns suggests that PvNRAMP1, −2, −3, −4 and −5 are involved in mineral uptake and mobilisation. The expression of PvNRAMP4 in the suspensor cells suggests a role in translocation of divalent cations from the endosperm to the embryo (Ishida et al., 2018). PvNRAMP9 is the only member of this family that shows differential expression in relation to the bean genotypes with contrasting seed Zn content (Astudillo‐Reyes et al. (2015). Two others putative NRAMP genes have been linked to the Fe and Zn contents of beans (Izquierdo et al., 2018). However, only one of these (PvNRAMP2) is annotated as a transporter (Table S1).

The NRAMP family proteins transport a wide range of metals, including Fe^2+^, Mn^2+^, Co^2+^ and Zn^2+^ (Nevo & Nelson, 2006). The rice (Yamaji et al., 2013) and barley NRAMP transporters (Wu et al., 2016) typically transport Mn^2+^ and other minerals. The CjNRAMP1 transporter from the legume *Crotalaria juncea* (Nakanishi‐Masuno et al., 2018) and MtNRAMP1 from *Medicago truncatula* (Tejada‐Jimenez et al., 2015) transport Cd^2+^ and Mn^2+^, as well as Fe^2+^ when expressed in heterologous systems. Zn is usually taken up as Zn^2+^ by the NRAMP transporters in the root epidermal cells. However, the IRT1‐like proteins can also transport Zn (Eide et al., 1996; Korshunova et al., 1999).

Several mechanisms of Fe uptake have been reported in legumes. These include the aforementioned soil acidification but also the secretion of secondary metabolites that serve to mobilise insoluble Fe^3+^. Arabidopsis roots secrete phenolic compounds such as flavins and coumarins to mobilise Fe from alkaline soils (Fourcroy et al., 2016; Robe et al., 2021; Rodriguez‐Celma et al., 2013). Similarly, the roots of the legume *Medicago truncatula* increase flavin synthesis to release insoluble Fe (Rodriguez‐Celma et al., 2013). Efflux transporters such as the ABC (ATP‐biding cassette) family transporter PDR9/ABC37 [pleiotropic drug resistance 9 (PDR9)/ATP‐binding cassette G37 (ABC37)] and the paralog phenolics efflux zero (PEZ) transporters serve to transport phenolic compounds into the rhizosphere (Fourcroy et al., 2016; Ishimaru et al., 2011). Of the 136 ABC transporters encoded in the bean genome, 26 proteins have a high identity to the PEZ1/ABCG37/PDR9 transporters (Table S1). Although there is a lack of direct evidence for phytosiderophore secretion from peanut roots, this species is able to take up the Fe^3+^ released by the phytosiderophore (PS) deoxymugineic acid (DMA) via a functional Fe^3+^–DMA transporter in the root epidermis. This process involves a member of the YS1/YSL family of transporters (Schaaf et al., 2004; Xiong et al., 2013). The ZIFL/TOM [Zinc‐Induced Facilitator‐Like (ZIFL)/Transporter Of Mugineic acid (TOM)] family of proteins is important in DMA secretion from roots, as well as PS and NA transport and Zn homeostasis (Ricachenevsky et al., 2015). The ZIFL transporters in rice and Arabidopsis show tissue‐specific expression, with overlapping expression patterns in response to changes in Fe and Zn levels (Ricachenevsky et al., 2011). Common bean has 10 putative ZIFL family members (Table S1), that may have specific roles. For example, PvZFL1 and PvZFL10 were found to have differential expression patterns in two bean genotypes with contrasting seed Zn concentrations (Astudillo‐Reyes et al., 2015).

## 6 ROOT‐TO‐SHOOT TRANSLOCATION VIA XYLEM

Root and shoot processes are closely co‐ordinated to maximise nutrient uptake. The networks of genes involved in the root responses of Strategy I and Strategy II plants to Fe‐deficiency have been characterised. However, the signalling pathways that control these processes at the whole plant level remain poorly understood, particularly in legumes where relatively few components involved in shoot‐to‐root communication of Fe deficiency have been identified.

Information concerning the Fe‐status of Strategy I shoots is transmitted to the roots to activate Fe‐starvation responses (García‐Mina et al., 2013). Similar processes are thought to be involved in the shoot‐dependent activation of Fe‐deficiency responses in Strategy II plants. Such responses require systemic signalling pathways that control root Fe uptake in response to Fe deficiency in the shoots. These systemic pathways involve auxin, Fe‐nicotianamine transporters and the IRON MAN (IMA) peptides (Garnica et al., 2018; Grillet et al., 2018; Kumar et al., 2017). The IMA peptides control Fe transport and signalling in Arabidopsis. Other potential Fe sensors include the rice HEMERYTHRIN MOTIF‐CONTAINING REALLY INTERESTING NEW GENE (OsHRZ1) and the ZINC‐FINGER PROTEIN 1 (OsHRZ2), which negatively regulate Fe homeostasis. The IMA peptides work together with BRUTUS (BTS), which negatively regulates Fe homeostasis by promoting the ubiquitin‐mediated degradation of bHLH105 and bHLH11 that are positive regulators of the Fe deficiency response. The IMA peptides sequester BTS and thus activate the Fe deficiency response by protecting bHLH105/bHLH115 from degradation (Li et al., 2021). A light‐dependent systemic signal transduction (phyB‐HY5‐FER) loop was found to regulate Fe uptake in tomato roots. This loop involves the phytochrome B (phyB)‐induced accumulation of the basic leucine zipper (bZIP) transcription factor called ELONGATED HYPOCOTYL 5 (HY5) in leaves and roots. HY5 movement from shoots to roots activates the expression of FER and increased Fe uptake (Guo et al., 2021).

The flow of chelated Fe‐ and Zn‐complexes through the plant requires transporters for xylem loading, as well as short‐ and long‐distance transport (Curie et al., 2009). The YSL family transporters are required for the movement of chelated Fe into the pericycle prior to loading into the xylem, where the pH of the xylem is likely to favour the generation of citrate‐Fe^3+^ complexes (Clemens, 2019; Palmer & Guerinot, 2009). This is supported by FERRIC REDUCTASE DEFECTIVE (FRD) and FRD‐like (FRDL) transporters that belong to the Multidrug and Toxic compound Extrusion (MATE) family and are responsible for citrate efflux into the root xylem (Yokosho et al., 2016). Other metal complexes such as Fe^3+^‐DMA and Zn^2+^‐DMA are found in the xylem sap of graminaceous (Nishiyama et al., 2012; Xuan et al., 2006) and non‐graminaceous plants (Suzuki et al., 2016) suggesting that metal complexes with organic molecules other than citrate play an important role in xylem transport.

Citrate efflux‐mediated transport by FRD/FRDL is coordinated with Fe efflux to the xylem. Three potential Fe transporters of the IRON REGULATED protein/Ferroportin/MULTIPLE ANTIBIOTIC RESISTANCE (IREG/FPN/MAR) family have been identified in Arabidopsis of which IREG1/FPN1 is likely to control Fe efflux from the pericycle to the xylem (Morrissey & Guerinot, 2009). Something similar appears to occur in legumes where ferric‐citrate complexes have been observed in soybean and high xylem citrate levels, dependent on the coordinated action of the GmFRD citrate transporters are required for efficient root‐to‐shoot Fe translocation (Rogers et al., 2009). The common bean genome encodes two putative PvIREG1/FPN1‐like and one MAR1‐like transporter as well as six FRD/FRDL proteins (Table S1).

Fe is required for the enzymes catalysing symbiotic nitrogen fixation in legumes. Transporters that maintain the Fe^3+^‐citrate levels in nodules have been reported in *Medicago truncatula*, *Lotus japonicus* and soybean. MtMATE67 is responsible for citrate efflux (Kryvoruchko et al., 2018), while Fe^2+^ uptake requires MtNRAMP1 (Tejada‐Jimenez et al., 2015). The soybean GmDMT1 transports Fe^2+^ (Kaiser et al., 2003) while GmMATE75, GmMATE79 and GmMATE87 are root citrate transporters (Zhou et al., 2019). The LjMATE1 citrate transporter provides Fe to the infection zone of *Lotus japonicus* nodules (Takanashi et al., 2013). Three MATE transporters have been linked to Fe and Zn homeostasis in beans (Izquierdo et al., 2018).

Members of the P_IB_‐type heavy metal ATPase (HMA) family are involved in loading Zn into the xylem, while the ZIP family transporters are mainly involved in Zn transport and homeostasis at the whole plant level (Ajeesh Krishna et al., 2020; Hussain et al., 2004). Several Quantitative Trait Loci (QTLs) have been linked to seed Fe and Zn concentrations in chickpea. These include HMA‐ and ZIP‐like transporters (Upadhyaya et al., 2016). The common bean genome encodes 13 HMA transporters (Astudillo et al., 2013; Astudillo‐Reyes, 2014), and 20 annotated PvZIP (Table S1). The expression levels of seven PvZIP genes have been analysed, revealing tissue‐specific expression patterns in response to Zn deficiency. PvZIP12, PvZIP13 and PvZIP16 genes are expressed in roots, leaves and pods. PvZIP12 is highly expressed in leaves at the vegetative stage, while PvZIP13 is expressed in leaves at flowering (Astudillo‐Reyes et al., 2015). These data indicate that tissue‐specific isoforms of ZIP‐like transporters are likely to be involved in Zn homeostasis in different tissues with combined transcriptional and genetic analysis highlighting PvZIP13, PvZIP18 and in particular PvZIP12 as strong candidates for mobilisation and transport of Zn to bean seeds (Astudillo et al., 2013). On the other hand, a meta‐QTL study linked PvZIP17 and PvZIP19 to seed Fe and Zn accumulation in a wider pool of common bean germplasm (Izquierdo et al., 2018). Of the 20 PvZIP genes identified in common bean, 11 are currently annotated as Zn transporters, while the rest are annotated as Fe/Zn transporters (Table S1). This classification is similar to Arabidopsis, in which eight of the 15 ZIP genes are induced by Zn deficiency (Thiébaut & Hanikenne, 2022). The large number of ZIP transporters may infer a degree of redundancy where IRT3, ZIP4, ZIP6 and ZIP9 have overlapping functions in Arabidopsis, each contributing to the maintenance of Zn homeostasis during seed development (Lee et al., 2021).

## 7 IRON AND ZINC PARTITIONING TO THE PHLOEM

The pH of the phloem sap favours the formation of NA‐Fe^2+^ and NA‐Zn^2+^ complexes, which can be transported throughout the plant (Clemens, 2019; Palmer & Guerinot, 2009). Arabidopsis has four nicotianamine synthase (NAS) genes that are differentially expressed in a tissue‐specific manner, in response to Fe deficiency (Klatte et al., 2009). Common bean has three genes encoding putative NAS proteins (Table S1). One of these may serve a role in seed Fe and Zn accumulation since it underlies a QTL for this trait (Izquierdo et al., 2018). The rice EFFLUX TRANSPORTER OF NA (ENA)‐like transporters were originally characterised via their NA transport capabilities in *Xenopus laevis* oocytes (Nozoye et al., 2011). More recently, functional studies have suggested a role for ENA1 in the long‐distance transport of Fe where ENA1 expression in shoots was limited to the xylem–phloem translocation interface at the root‐to‐shoot junction (Nozoye et al., 2019). Recently, two members of the nitrate/peptide transporter family (NAET1 and NAET2) were shown to be important for the NA efflux required for Fe translocation to Arabidopsis seeds (Chao et al., 2021). These proteins were shown to be NA transporters by heterologous expression in yeast cells engineered to synthesise NA. Although the single *naet1 or naet2* Arabidopsis mutants had wild‐type phenotype, the *naet1naet2* double mutants exhibited chlorosis and embryo development defects combined with reduced seed NA contents and a severe reduction in the Fe content of the sink tissues (flowers, seeds, young leaves). Only one putative NAET‐like transporter has been identified in the common bean genome. This may represent a useful target in future biofortification strategies (Table S1).

YSL transporters are a subfamily of the Oligopeptide Transporter (OPT) family that belongs to the major facilitator superfamily. The YSL transporters are required for the import of chelated Fe into the roots and also radial transport in the roots before translocation to the shoots (Araki et al., 2011; Schaaf et al., 2004; Xiong et al., 2013; Zheng et al., 2011). YSL proteins mediate long‐distance trafficking of metal‐NA complexes, and they are important in remobilising intracellular Fe and Zn reserves. The OPT genes show organ‐specific and tissue‐specific expression patterns (Su et al., 2018). These transporters participate in the transfer of Fe from the xylem to the phloem and regulate both shoot‐to‐root Fe signalling for Fe/Zn/Mn status, and Fe remobilisation from mature to developing tissues (García et al., 2013; Ishimaru et al., 2010; Mendoza‐Cózatl et al., 2014; Zhai et al., 2014). The import and storage of Fe and Zn for processes such as photosynthetic and mitochondrial electron transport in leaves and reproductive organs (see Vigani et al. (2019 and references therein). This process involves ferroportin (FPN; Kim et al., 2021) and the mitochondrial Fe transporter (MIT; Jain et al., 2019), which co‐ordinate import with the assembly of cofactors, so as to avoid the uncontrolled generation of ROS (reactive oxygen species; Lopez‐Millan et al., 2016).

Studies using labelled ^55^Fe supplied to castor bean seedlings suggested that Fe translocation was mediated via chelation to proteins. Much of the ^55^Fe in the phloem sap was recovered in the protein fraction following size exclusion chromatography. This Fe was bound to a low molecular weight protein of the Late Embryogenesis Abundant family subsequently named ITP (Iron Transport Protein; Kruger et al., 2002). Such data led to the hypothesis that while NA serves as a shuttle facilitating the translocation of Fe into and out of the phloem, long‐distance transport within the phloem requires a peptide chelator (Morrissey & Guerinot, 2009).

## 8 SEED MINERAL LOADING

Since Fe and Zn are required for embryogenesis (Connorton et al., 2017), seed development is contingent on the activity of YSL/OPT transporters (Mari et al., 2020; Senoura et al., 2017; Stacey et al., 2008; Zang et al., 2020). These transporters participate in the unloading of NA‐metals from the phloem into the seeds for subsequent use in the cells or storage, processes that require the coordinated action of the Vacuolar‐Iron Transporter‐Like (VTL; Eroglu et al., 2019; Kim et al., 2006; Mari et al., 2020; Ram et al., 2021; Zhang et al., 2012), NRAMP (Bastow et al., 2018; Lanquar et al., 2005; Mari et al., 2020; Mary et al., 2015) and metal tolerance protein (Chu et al., 2017) transporters. Comparisons of the expression profiles of these genes from common bean pods with differential Zn seed contents, led Astudillo‐Reyes et al. (2015) to speculate that the observed variations were caused by differences in the Zn storage capacity of organs other than seeds.

The common bean genome encodes 19 putative YSL/OPT proteins (Table S1) of which four are annotated as metal‐NA YSL transporters and the rest as OPT transporters. Astudillo‐Reyes et al. (2015) described nine YSL proteins in two navy bean genotypes although two of these are not recorded in the Phytozome database. Studies using a combination of QTL mapping, SNP analysis and gene expression analysis implicated YSL transporters in loading Fe and Zn into chickpea seeds (Upadhyaya et al., 2016). The common bean genome has nine putative NRAMP and 11 VTL sequences. Four of the 15 members described by Astudillo‐Reyes et al. (2015) do not appear to be VITs (Table S1).

The differential distribution and accumulation of Fe, and Zn, in the different seed cell layers of the seeds is likely to result from variations in transporter function. About 50% of these minerals found in Arabidopsis seeds are concentrated in the vacuoles of the radicle and cotyledons of the embryo (Rehman et al., 2021). On the contrary, most of the Fe (71%–94%) is stored in the cotyledons of common bean seeds with the remainder distributed between the seed coat (3%–26%) and the embryo axis (1%–4%; Ariza‐Nieto et al., 2007; Zeffa et al., 2021). Similarly, approximately 90% of the Zn is stored in the cotyledons while the rest is distributed between the seed coat (~6%) and the embryo axis (~5%; Zeffa et al., 2021).

Fe and Zn must be stored in a stable form that can be remobilised during germination. Between 15% and 30% of the Fe is bound to ferritin in common beans seeds, while 70%–85% is in the form of non‐ferritin‐bound Fe, possibly bound to phytate (Hoppler et al., 2009). Phytate and its derivatives are the most abundant (65%–85%) storage form of phosphorus in seeds which is needed for important cellular functions during germination and seedling development (Madsen & Brinch‐Pedersen, 2020). It also seems likely that phytate, or a derivative, forms Zn complexes in seeds (Neal et al., 2013; Zhang et al., 2020). The capacity of phytates to bind minerals means they act as antinutrients in the human diet as the human intestine lacks phytate‐degrading enzymes required to allow absorption of phytate chelated minerals (Iqbal et al., 1994). Phytate distribution is similar to that of Fe and Zn in common bean seeds with between 94 and 98% of the total localised to the cotyledons (Ariza‐Nieto et al., 2007). A recent review (Colombo et al., 2020) has discussed how the accumulation of phytate in the vacuole is associated with members of the MRP/ABC [Multidrug Resistance‐associated Protein (MRP) ATP‐binding cassette (ABC)] transporter subfamily in a wide variety of plant species, including Arabidopsis, rice, soybean and common bean (Cominelli et al., 2018). However, these are not the only transporters and enzymes associated with phytate accumulation (Cominelli et al., 2020b, 2020c).

Ferritin proteins (Fer) are found mainly in plastids, but also in the mitochondria (Chiou & Connor, 2018). They are comprised of a polymeric shell that is usually composed of 24 identical polypeptides surrounding an Fe core (Zielinska‐Dawidziak, 2015). Fer–Fe complexes are an excellent bioavailable source of Fe because the proteins are denatured at the high temperatures obtained during cooking to release free Fe (Hoppler et al., 2008; Moore et al., 2018). In one study, up to 90% of Fe was reported to be ferritin‐bound in soybean seeds (Ambe et al., 1987). However, other reports suggest lower levels of ferritin‐Fe in pulse legumes which ranged from 18% to 49% in soybeans, 52%–62% in dry peas, 69% in lentils and 15%–29% in common beans (Hoppler et al., 2008, 2009; Lonnerdal, 2009). Although soybean ferritin has been the target of many studies on seed Fe accumulation, it has not been evaluated in other legumes (Sperotto et al., 2018), although the *FER* genes have been a target for genetic engineering to increase bioavailable Fe in common bean seeds (Table S1; Sperotto & Ricachenevsky, 2017). However, not all *FER* genes are expressed in the seeds (Ravet et al., 2009) and some are responsible for the control of Fe‐induced oxidative stress and regulation of Fe homeostasis in other tissues (Parveen et al., 2016; Reyt et al., 2015). More detailed studies are therefore required to determine which *FER* genes are the best targets for manipulation in common bean seeds. Other studies have indicated that while high concentrations of Fe (up to 0.5 mg/kg) accumulate around the provascular tissues in *P. vulgaris*, ferritin is mainly accumulated in the amyloplasts of the embryonic cells (Cvitanich et al., 2010).

## 9 TRANSCRIPTIONAL REGULATION OF IRON AND ZINC HOMEOSTASIS

Although Fe and Zn homeostasis are controlled at multiple levels, much emphasis has been placed on the regulation of gene expression (Gao & Dubos, 2021; Velez‐Bermudez & Schmidt, 2021). The expression of the *IRT1* and *FRO2* genes is regulated by the bHLH29‐like transcription factor (TFs) called FER‐LIKE IRON DEFICIENCY INDUCED TRANSCRIPTION FACTOR (FIT). FIT activity is controlled by the interaction of many other bHLH TFs (bHLH38, bHLH39, bHLH100 and bHLH101, bHLH18, bHLH19, bHLH20 and bHLH25). A second layer of positive regulation involves bHLH105, bHLH34, bHLH104 and bHLH115. There are a range of negative regulators such as the bHLH47, bHLH11 and bHLH121. Three bHLH TFs have been characterised in soybean with respect to Fe and Zn homeostasis (Li et al., 2018; Xu et al., 2017). Two of these exhibit tissue‐specific expression patterns in the root and nodule where their expression is primarily controlled by Fe availability (Li et al., 2018). While transcriptomic approaches have been previously used to identify the transcriptional regulators of Fe and Zn uptake and transport in common beans, reports were limited to biological activities (Santos et al., 2013) or the information on transcriptional regulators is scarce (Astudillo‐Reyes et al., 2015). Homologs of some of the potential regulators can be identified through a BLAST search of the Phytozome database (Table S1). As discussed previously, the transcriptional control of Fe uptake is partially regulated by the E3 ligase activity of BRUTUS/HRZ‐like proteins, which target some of the bHLH TFs for degradation (Hindt et al., 2017; Kobayashi et al., 2013).

Three putative BRUTUS/HRZ‐like proteins were identified in common bean (Sperotto & Ricachenevsky, 2017) but only two appear to be true BRUTUS/HRZ‐like proteins (Table S1). This regulatory mechanism is complex because BRUTUS can interact with small peptides such as IMA/FEP [IRON MAN (IMA)/FE UPTAKE‐INDUCING PEPTIDE (FEP)]. FEP is considered to be an important positive regulator of Fe acquisition, acting as a phloem‐mobile signal (Grillet et al., 2018; Hirayama et al., 2018; Kobayashi et al., 2021) that maintains Fe homeostasis in Arabidopsis (Li et al., 2021). The IMA/PEP peptides sequester BTS and thus activate the Fe deficiency response by protecting bHLH105/bHLH115 from degradation (Li et al., 2021). To date, IMA/FEPs have been identified in several different species, including soybean (Kobayashi et al., 2021). While none of these peptides are expressed in seeds, the overexpression of IMA/FEPS results in increased Arabidopsis and rice seed Fe contents (Grillet et al., 2018; Hirayama et al., 2018; Kobayashi et al., 2021).

A genome‐wide association study recently identified a further ubiquitin‐protein E3 ligase associated with seed Zn contents. The Phvul.001G233500 gene encodes an SDIR1‐like protein that is involved in the regulation of abscisic acid signalling (Caproni et al., 2020). In addition to the bHLH TFs, several bZIP TFs are induced by Zn deficiency and are involved in the control of the expression of ZIP transporters (Castro et al., 2017; Lilay et al., 2020; Sinclair & Kramer, 2012). They have also recently been described as Zn sensors (Lilay et al., 2021). Three common bean bZIP TFs (Table S1) are differentially expressed in response to mineral deficiency. PvbZIP1 is highly expressed in leaves and pods while the other two (PvbZIP2 and 3) are associated with QTLs for seed Zn accumulation (Astudillo et al., 2013). However, no differences were reported in the expression of these TFs in comparisons of pods from two bean genotypes with contrasting seed Zn content (Astudillo‐Reyes et al., 2015).

The Arabidopsis INO (INNER NO OUTER) is a member of the YABBY TF family. INO negatively regulates NRAMP expression by targeting the promoter region to regulate seed Fe loading. Four INO TFs are present in the common bean genome (Table S1). Tissue‐specific regulation of the expression of these genes during embryogenesis and seed development could have great biotechnological potential (Sun et al., 2021). Other regulators such as nitric oxide (NO) may be involved in the control of the expression of the enzymes and proteins modulating Fe homeostasis (Tewari et al., 2021).

## 10 BIOAVAILABILITY OF FE AND ZN

Biofortified legumes and other crops have so far not clearly demonstrated the relationship between enhanced dry seed content of Fe and Zn and increased bioavailability of target elements. Whilst there are successes with Fe and Zn biofortified legumes (Tako et al., 2011, 2015), it has not always been possible to demonstrate differences in bioaccessibility (fraction of micronutrients available for absorption by the intestinal mucosa) or bioavailability (fraction of micronutrients that crosses the intestinal barriers and is available to the body) between biofortified and non‐biofortified legumes (Vaz‐Tostes et al., 2016). Glahn and Noh (2021) highlighted the lack of evidence to support the assumption of a positive association between higher bean seed Fe and increased Fe absorption, and the need to focus on bioavailability traits.

The bioaccessibility/bioavailability of Fe and Zn can be determined by different approaches, including animal models, human studies and in vitro methods (Dias et al., 2018; Etcheverry et al., 2012). For common bean, techniques such as in vitro digestion and absorption/transport to Caco‐2 cells (Ariza‐Nieto et al., 2007) or capacity to cross a low molecular cut off dialysis membrane (Coelho et al., 2021; Huertas et al., 2022) have been adopted as proxies for bioavailability. However, solubility and dialysability methods have a tendency to overestimate bioavailable Fe but have the advantage of also quantifying bioavailable Zn (Dias et al., 2018). All current methods suffer from a lack of standard procedures. Hence a combination of in vitro digestion (e.g. standardised INFOGEST protocol (Brodkorb et al., 2019) and the Caco‐2 absorption model is often recommended (Bohn et al., 2018) although not always available to non‐specialist labs that lack the capacity to maintain human cell lines.

The bioavailability of Fe (and to a lesser extent of Zn) is strongly influenced by inhibitors and enhancers. The main inhibiting factors are phytic acid (PA), tannins, dietary fibre and calcium. PA is the main storage form of phosphate in cereal and legume grains. It forms insoluble complexes with Fe, especially under pH conditions (pH 6–7) found in the small intestine (Ferruzzi et al., 2020). In contrast, ascorbic acid exerts a positive effect on bioavailability because it reduces Fe^3+^ to Fe^2+^, the soluble and absorbable form of Fe. Zn bioavailability is also dependent on components present in the intestinal lumen. PA and nucleic acids decrease Zn absorption, while animal proteins such as from beef, eggs and cheese exert a positive effect on Zn absorption possibly via chelation. Although Fe has been reported to inhibit Zn absorption, this is only evident in the absence of a food matrix and with high Fe to Zn ratios that is of 25:1 (Etcheverry et al., 2012).

Given the strong inhibitory influence of PA on Fe bioavailability, reduction of PA in seeds (Petry et al., 2013, 2014), and dephytinisation strategies that involve the activation of endogenous phytase enzymes or the addition of exogenous phytase may have positive effects on mineral bioavailability (Nielsen et al., 2013). However, it would be necessary to maintain a balance between phytase activity and the amount of PA to avoid the adverse gastrointestinal symptoms associated presumably to increased stability of lectin phytohemagglutinin L (PHA‐L) in the low‐PA beans (Cominelli et al., 2020a; Petry et al., 2016).

Soaking, germination or fermentation of cereal/legume‐based foods removes about 50% the PA (Gibson et al., 2010) and it is assumed that such a reduction will lead to an improvement in mineral absorption in high phytate containing foods. However, the magnitude of any increase in absorption may be difficult to predict due to the presence of other antinutrients. For example, some polyphenols have been reported to promote Fe release and absorption (e.g. kaempferol, catechin, kaempferol 3‐glucoside and 3,4‐dihydroxybenzoic acid), while others inhibit (e.g. quercetin, myricetin, quercetin 3‐glucoside and myricetin 3‐glucoside) these processes (Cárdenas‐Castro et al., 2020; de Figueiredo et al., 2017; Hart et al., 2017; Laparra et al., 2008). Many classes of polyphenol have been isolated from beans (Yang et al., 2018), but the key regulatory genes involved in the production of these specialised secondary metabolites in the seed are largely unknown.

Cooking is very important to increase bioavailability. Heat‐treated beans have reduced effects of toxic and anti‐nutritional substances, and at the same time increased protein digestibility and general palatability (de Oliveira et al., 2018). However, it remains difficult to predict how specific food processing methods and cooking time will affect mineral quantity and bioavailability (Cárdenas‐Castro et al., 2020; Chinedum et al., 2018; Rousseau et al., 2020; Wiesinger et al., 2018).

Ferritin has received considerable attention as potential target for increasing Fe bioavailability in beans as the ferritin content of pulses is higher than that of cereal grains. The ferritin‐bound Fe differs between varieties in common bean ranging from approximately 15% to 30% (Hoppler et al., 2008; Hoppler et al., 2009). Ferritin is not fully stable in the low pH environment of the digestive tract, leading to the release of free Fe from the ferritin protein (Kalgaonkar & Lonnerdal, 2008). Similarly, cooking, particularly boiling, of legume grains destroys ferritin which is no longer detectable after 50 min of processing (Hoppler et al., 2008). However, different ferritin subunits have different stability profiles with the H‐2 subunit more stable and less susceptible to proteolysis than the H‐1 subunit (Masuda et al., 2001). Ferritin subunit composition is species specific; for example, pea ferritin contains more H‐2 subunits than soybean ferritin and therefore may be less prone to degradation. Similarly, the ferritin subunit composition is different in black bean and soybean, which have ratios of H1:H2 subunits of 2:1 versus 1:1, respectively (Deng et al., 2010). In addition, the binding of anthocyanins to soybean ferritin increased the stability of the protein in stimulated intestinal fluid studies (Deng et al., 2011). Ferritin is taken up by endocytosis into the enterocytes of the intestines. Therefore, uptake does not involve transport systems for ferrous Fe or haem Fe (Pereira et al., 2013; San Martin et al., 2008). Protecting ferritin from digestion in the low gastric pH conditions of the intestines has been suggested as strategy for the treatment of Fe deficiency (Perfecto et al., 2018) and the observation that different species tolerate widely differing ferritin subunit distribution suggests that breeding for optimal ferritin subunit distribution could improve Fe bioavailability in beans without impairing the physiological function of ferritin *in planta*.

## 11 GENETIC RESOURCES AND BIOFORTIFICATION STRATEGIES FOR COMMON BEAN

The genetic variation in seed Fe and Zn accumulation found in the seeds of different common bean genotypes provides a basis for the selection of improved varieties with enhanced biofortification characteristics. Several studies have been performed using bean germplasm from the Andean and Mesoamerican gene pools (Kwak & Gepts, 2009; Schmutz et al., 2014). Although many QTL have been associated to these traits (Izquierdo et al., 2018; Jha & Warkentin, 2020; Losa et al., 2022; Philipo et al., 2021 and references therein), relatively few genetic markers or candidate genes have been identified (Refs across this review).

The first chromosome‐scale bean reference genome was provided by the Department of Energy's Joint Genome Institute (Schmutz et al., 2014). Recent advances in bean genomics, such as the Illumina BARCBean6K_1 BeadChip with >5000 single nucleotide polymorphisms (SNPs; Song et al., 2015), and genotyping‐by‐sequencing dense SNP genetic maps (Schröder et al., 2016) have facilitated allelic screening using germplasm collections worldwide (Table S2). Genome‐wide association studies (GWAS), which are considered to be the next step after QTL mapping, have already combined SNP markers and phenotyping to uncover the genetic basis of mineral (including Fe and Zn) accumulation (Table S2 and references therein). The *P. vulgaris* Gene Expression Atlas (PvGEA) provides information on gene expression patterns in different tissues (O'Rourke et al., 2014). However, PvGEA is based on a single bean genotype, that is cv. Negro Jamapa (Mesoamerican genotype) and further datasets from the diversity of germplasm grown globally will significantly enhance the value of this resource. An example of efforts to enrich PvGEA include the assembly of RNA‐Seq and GWAS to narrow identify target genes associated to pod and seed traits in common bean (Di Vittori et al., 2021; McClean et al., 2018).

Genetic engineering and gene editing approaches offer rapid alternatives to standard plant breeding methods. However, such approaches require the identification of precise genetic targets that control bottlenecks in Fe/Zn allocation and accumulation in seeds, as well as bioavailability. Such strategies will need to identify genes and mechanisms associated with the transport of Zn and Fe from the soil to the beans. Many of the genes highlighted in the above discussion and summarised in Table S1, have the potential to have direct or indirect effects on seed Fe/Zn accumulation. A knowledge of the tissue‐specific expression of target genes that regulate uptake, allocation or systemic responses is required to modify whole‐plant metal homeostasis and deliver seed Fe and Zn accumulation.

Our current knowledge of the genes and processes involved in Fe and Zn uptake and storage in different plant species has identified potential targets for marker‐assisted selection and genetic improvement. Genetic manipulation studies have already led to some successes in increasing the Fe and Zn contents of key crops. For example, in field trials, cassava lines co‐expressing a mutated Arabidopsis Fe transporter (IRT1) and ferritin (FER1) were shown to accumulate more Fe (7–18 times higher) and Zn (3–10 times higher) than controls. These IRT1 + FER1 lines could provide 40%–50% of the estimated average requirement (EAR) for Fe and between 60% and 70% of the EAR for Zn in 1‐ to 6‐year‐old children and nonlactating, nonpregnant West African women (Narayanan et al., 2019).

Several studies have focused on decreasing phytate concentrations (Cominelli et al., 2020b and references therein). Other studies have sought to increase ferritin‐Fe accumulation (Sperotto et al., 2018 and references therein). The latter approach appears to have given the most promising results. Furthermore, like pea seeds (Moore et al., 2018), common bean seeds accumulate ferritin in different intracellular compartments to those that accumulate phytate (Cvitanich et al., 2010), so the two approaches are compatible. A recent analysis of VTL transporters has shown that they could play an important role in ensuring the optimal compartmentalisation of Fe (Eroglu et al., 2019).

Transformation protocols have been standardised for several legumes (Bhowmik et al., 2021; Table S1). However, common bean is a recalcitrant crop for transformation and the low capacity for in vitro regeneration is a particular barrier to success (Hnatuszko‐Konka et al., 2014). Stable transformation of common bean has been achieved using biolistic‐mediated transformation of meristematic tissues but with very low frequencies (<1%; Aragão et al., 1998; Bonfim et al., 2007; Kim & Minamikawa, 1996; Ramirez Rivera et al., 2016). The transformation frequency is a cultivar‐dependent trait (Mukeshimana et al., 2013). Agrobacterium‐mediated transformation and shoot regeneration through somatic embryogenesis has been successfully implemented recently but with low transformation frequencies (0.5%–2.5%) indicating that this technology is far from routine (Solís‐Ramos et al., 2019; Song et al., 2020).

## 12 CONCLUSIONS AND PERSPECTIVES

While dietary supplements and food fortification can be effective solutions to Fe and Zn deficiencies, such strategies fail to reach target populations including the urban poor and those in rural areas, are not sustainable economically in the long term, especially in low‐income countries. Interest in using beneficial soil microbes as an agronomic strategy to improve mineral uptake and accumulation in dietary food grains and legumes has increased in recent years. However, its potential is still to be explored across crops, ecologies and farming systems (Roriz et al., 2020; Singh & Prasanna, 2020). An alternative strategy to which the plant science and breeding sectors can make a significant contribution is the development of biofortified cultivars that accumulate essential mineral nutrients. While genome editing techniques allow precise modification of plant genomes (Menz et al., 2020) and biofortification through the genetic improvement of crops can be effective, plant breeding for improved nutritional trains remains a relatively slow process and must, if it is to succeed, encompass the bioaccessibility and bioavailability aspects of Fe and Zn as part of the phenotyping process.

Efforts to increase Fe and Zn in cereals using biotechnological approaches have achieved some success (Majumder et al., 2019; Table 1; Stanton et al., 2022; Table S1). Gene‐editing approaches, mainly using Clustered Regularly Interspaced Short Palindromic repeats/CRISPR‐Associated Protein 9 (CRISPR/Cas9)‐mediated targeted modifications have proved to be useful (Achary & Reddy, 2021; Che et al., 2019, 2021; Ibrahim et al., 2021). While common bean is an important target for biofortification using such approaches, it is not readily accessible to current biotechnological methods. Hence, classical breading approaches are likely to be the most successful in the short term, especially in developing countries.

Our knowledge of the mechanisms by which Fe and Zn accumulate in the grains of legumes remains incomplete. The genes encoding proteins involved in mineral translocation and accumulation exist in large gene families. Hence, the identification of specific function on the basis of sequence homology alone remains a significant challenge. Well‐designed transcriptional profiling experiments that take advantage of diverse germplasm will provide a valuable resource in the elucidation of important genes in legumes. However, a step change in fundamental knowledge concerning the kinetics of grain mineral accumulation and the integration of these processes with other developmental processes, is required to drive significant advances in biofortification. Limitations in methodology, particularly for common bean and other legume species must be overcome. While technical advances are frequent, the applicability of techniques such as gene editing to common bean and other species and genotypes remains problematic (Bhowmik et al., 2021).

At a global level the interplay between agriculture, climate change, GHG emissions, food security and nutrition are the topic of considerable debates such as conventional versus regenerative agriculture, circular versus linear production chains and livestock versus plant‐based foods. Within this context, the potential for plant‐based foods in minimising the environmental impact of agriculture and providing a comparatively inexpensive source of appropriate nutrition, cannot be understated. Especially for developing countries, beans and other legumes provide an attractive alternative protein source for livestock, which are responsible for almost 15% of total anthropogenic GHSs (Grossi et al., 2019). Furthermore, global legume‐rhizobial symbioses are estimated to fix 21Mt of nitrogen annually, representing approximately one tenth of the ammonia applied annually synthesised by the Haber‐Bosch process (Foyer et al., 2016). As estimated CO_2_ emissions for ammonia production are 7.2 kg/kg (Chai et al., 2019), this represents an equivalent CO_2_ saving of over 150 Mt per annum equivalent to approximately half of the annual agricultural GHG emissions of East Africa (Tongwane & Moeletsi, 2018). There is therefore an urgent need to improve key traits in grain legumes not only for populations that rely heavily on them as food sources and where mineral deficiencies are widespread but also for other populations that seek to move away from meat‐dependent diets to reduce environmental impacts of agriculture and where specific groups in particular have significant mineral deficiencies. Within this context, we have discussed current knowledge with a particular focus on common bean and highlighted the significant global efforts to develop improved grain legumes with enhanced Fe and Zn contents. Increases in the levels of Fe and Zn of up to approximately 130 and 60 mg/kg have been achieved in biofortified crops (Kimani & Warsame, 2019). These levels compare favourably with those found in animal products (15–110 mg/kg Fe, 23–170 mg/kg Zn dry weight basis; Gerber et al., 2009). Bioavailability differs significantly dependent on dietary source. For example, Fe bioavailability in a meat‐based diet is estimated at 15%–18% and high levels of ascorbate additionally aid absorption, on the contrary Fe in a tuber/cereal‐based diet is much lower at 5% (Hurrell & Egli, 2010). This is further increased because dry grains contain little or no ascorbate (De Tullio & Arrigoni, 2007). The intensity of current research effort is therefore likely to ensure the success of current bean biofortification programmes.

## 14 CONFLICT OF INTEREST

The authors have stated explicitly that there are no conflicts of interest in connection with this article.

15

## Supporting information


Table S1

Table S2

## Data Availability

The data supporting this review is openly available in the references and DOIs provided across the manuscript.
